# Supplementary material for: Coping as a Mediator and Moderator between Psychological Distress and Disordered Eating Behaviors and Weight Changes during the COVID-19 Pandemic
Source: Int J Environ Res Public Health. 2023 Jan 31;20(3):2504. doi: 10.3390/ijerph20032504 (PMC9915561; doi:10.3390/ijerph20032504)
Supplement: Supplementary file 1 [file ijerph-20-02504-s001.zip › ijerph-2161138-supplementary.pdf]

Supplementary material

Y<br>Coping strategy	X<br>Psych. Distress / Disordered eating	General Interpretation of the moderator effect of Y in the causal relationship<br>X → Weight change<br>(using model estimates and graphical representations)
Planning	Anxiety	**(I)** For lower levels of Y, higher levels of X are associated with higher weight gains. However, increasing simultaneously X and Y is associated with weight loss. Thus, as levels of Y increase, the harmful effect of X on weight decreases. This means that these coping strategies benefited people with anxiety, stress, and disordered eating (global, weight concern, restraint) symptomatology to preserve or lose weight, in general.
Positive reframing	Anxiety<br>Stress<br>EDE global<br>Weight concern<br>Restraint	
Acceptance	Restraint	**(II)** The simultaneous increase of restraint and acceptance is associated with weight loss. This means that this coping strategy helped people with high levels of restraint to lose weight.
Denial	Weight concern	**(III)** The simultaneous increase of X and Y is associated with weight gain. This means that these coping strategies were harmful to people with disordered eating (global, weight and shape concerns).
Self-distraction	EDE global<br>Shape concern	
	Restraint	**(IV)** For low levels of self-distraction, greater restraint is associated with weight loss. The simultaneous increase in self-distraction and restraint is associated with weight gain. Thus, as self-distraction increases, greater restraint becomes associated with greater weight gain. This means that this coping was harmful to people with high levels of restraint.
Substance use	EDE global<br>Eating concern<br>Weight concern	**(V)** In general, both eating disorders (global, eating and weight concerns) and substance use are associated with weight gain. However, the simultaneous increase of both variables attenuates the harmful effect of on the weight. This means that substance use benefited people with high levels of disordered eating (global, eating and weight concerns).[1]
	Restraint	**(VI)** Higher substance use is associated with greater weight gain. However, the simultaneous increase in substance use and restraint is associated with weight loss. Thus, as restraint increases, greater substance use becomes associated with greater weight loss. This means that substance use benefited people with high levels of restraint.[1]

MODEL OUTPUTS

Coping	Direct effect	Mediation effect	Moderator effect
Active coping	--		
Planning			-- (anxiety)
Use of instrumental support	--		
Use of emotional support			
Religion			
Positive reframing	--		-- (anxiety, stress, DE)
Self-blame	++		
Acceptance			-- (restraint)
Venting			
Denial	++		++ (DE)
Self-distraction	++		++ (DE)
Behavioral disengagement	++	++	
Substance use	++		++ (DE)
Humor			

Coping	Direct effect	Mediation effect	Moderator effect
Active coping	--		
Planning			-- (anxiety)
Use of instrumental support	--		
Use of emotional support			
Religion			
Positive reframing	--		-- (anxiety, stress, DE)
Self-blame	++		
Acceptance			-- (restraint)
Venting			
Denial	++		++ (DE)
Self-distraction	++		++ (DE)
Behavioral disengagement	++	++	
Substance use	++		++ (DE)
Humor			

```
# ANXIETY x ACTIVE COPING

            Estimate Std. Error z value Pr(>|z|)
(Intercept) -0.12623    0.16912  -0.746  0.45545
anx          0.53774    0.18272   2.943  0.00325 **
cope2        0.01366    0.10522   0.130  0.89669
anx:cope2   -0.24215    0.11157  -2.170  0.02998 *
---

# ANXIETY x PLANNING

            Estimate Std. Error z value Pr(>|z|)
(Intercept) -0.03571    0.16225  -0.220  0.82581
anx          0.53785    0.17342   3.101  0.00193 **
cope6       -0.03580    0.10011  -0.358  0.72063
anx:cope6   -0.32478    0.12443  -2.610  0.00905 **
---

# STRESS x USE OF INSTRUMENTAL SUPPORT

             Estimate Std. Error z value Pr(>|z|)
(Intercept)  -0.19711    0.18803  -1.048 0.294500
stress        0.47393    0.14081   3.366 0.000764 ***
cope6         0.01381    0.11579   0.119 0.905062
stress:cope6 -0.24279    0.09788  -2.481 0.013119 *
---

# EDE GLOBAL x POSITIVE REFRAMING

            Estimate Std. Error z value Pr(>|z|)
(Intercept) -0.05916    0.15804  -0.374 0.708152
ede          0.29804    0.08670   3.438 0.000587 ***
cope6       -0.06304    0.10318  -0.611 0.541199
ede:cope6   -0.13605    0.06098  -2.231 0.025675 *
---

# WEIGHT CONCERN x POSITIVE REFRAMING

             Estimate Std. Error z value Pr(>|z|)
(Intercept)  -0.06836    0.15882  -0.430 0.666886
ede_wc        0.23622    0.06803   3.472 0.000516 ***
cope6        -0.06494    0.10299  -0.631 0.528365
ede_wc:cope6 -0.10472    0.04723  -2.217 0.026604 *
---

# RESTRAINT x POSITIVE REFRAMING

            Estimate Std. Error z value Pr(>|z|)
(Intercept)  0.08121    0.15182   0.535  0.59273
ede_r        0.20630    0.08949   2.305  0.02115 *
cope6       -0.03131    0.09675  -0.324  0.74626
ede_r:cope6 -0.17452    0.05614  -3.109  0.00188 **
---
# RESTRAINT x ACCEPTANCE

            Estimate Std. Error z value Pr(>|z|)
(Intercept)  0.03321    0.16768   0.198   0.8430
ede_r        0.14693    0.09330   1.575   0.1153
cope8        0.01569    0.10441   0.150   0.8806
ede_r:cope8 -0.13578    0.05925  -2.292   0.0219 *
---

# WEIGHT CONCERN x DENIAL
```

```
              Estimate Std. Error z value Pr(>|z|)
(Intercept)   -0.08011    0.11037  -0.726   0.4679
ede_wc         0.01889    0.05001   0.378   0.7057
cope10        -0.20425    0.19461  -1.050   0.2939
ede_wc:cope10  0.14645    0.05615   2.608   0.0091 **
---
```

EDE GLOBAL x SELF-DISTRACTION

```
            Estimate Std. Error z value Pr(>|z|)
(Intercept) -0.06881    0.13785  -0.499   0.6177
ede         -0.05744    0.09801  -0.586   0.5578
cope11      -0.05631    0.11675  -0.482   0.6296
ede:cope11   0.12775    0.06065   2.106   0.0352 *
---
```

SHAPE CONCERN x SELF-DISTRACTION

```
              Estimate Std. Error z value Pr(>|z|)
(Intercept)   -0.08308    0.13780  -0.603   0.5466
ede_sc        -0.02008    0.07529  -0.267   0.7896
cope11        -0.11988    0.12037  -0.996   0.3193
ede_sc:cope11  0.11624    0.04854   2.394   0.0166 *
---
```

RESTRAINT x SELF-DISTRACTION

```
             Estimate Std. Error z value Pr(>|z|)
(Intercept)   0.03805    0.13320   0.286   0.7751
ede_r        -0.22967    0.09307  -2.468   0.0136 *
cope11        0.04678    0.10919   0.428   0.6683
ede_r:cope11  0.13106    0.06010   2.181   0.0292 *
---
```

EDE GLOBAL x SUBSTANCE USE

```
            Estimate Std. Error z value Pr(>|z|)
(Intercept) -0.23331    0.09566  -2.439 0.014726 *
ede          0.15777    0.05661   2.787 0.005321 **
cope13       0.92115    0.27568   3.341 0.000834 ***
ede:cope13  -0.23626    0.08867  -2.665 0.007710 **
---
```

EATING CONCERN x SUBSTANCE USE
